# Supplementary material for: 9.2%-efficient core-shell structured antimony selenide nanorod array solar cells
Source: Nat Commun. 2019 Jan 10;10:125. doi: 10.1038/s41467-018-07903-6 (PMC6328536; doi:10.1038/s41467-018-07903-6)
Supplement: Supplementary file 1 — Supplementary Information [file 41467_2018_7903_MOESM1_ESM.pdf]

Supplementary information

9.2%-efficient core-shell structured antimony selenide nanorod array solar cells

Li et al.

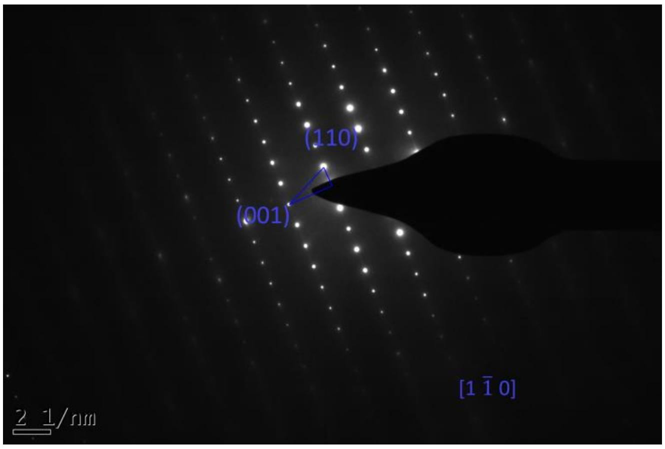

Supplementary Figure 1 SAED pattern of the Sb_2Se_3 nanorod arrays. Diffraction spots of the SAED patterns were indexed to (001) and (101) planes and thus [1$\bar{1}$0] zone axis was identified. It is different with the SAED pattern shown in Figure 1f due to the different observation angles.

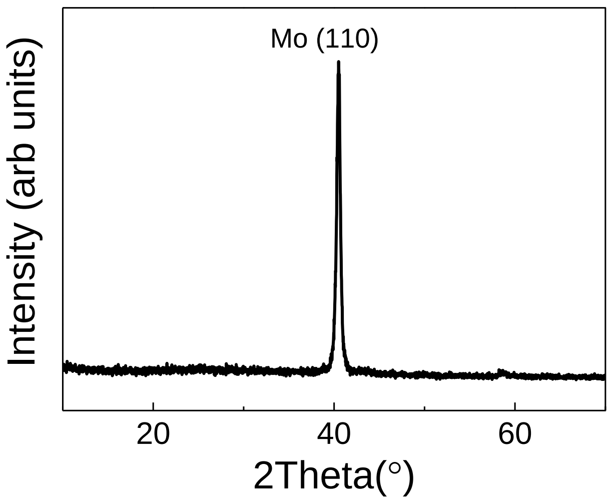

Supplementary Figure 2 XRD pattern of the Mo back contact layer. It displays a strong (110) orientation preference (JCPDS: 42-1120).

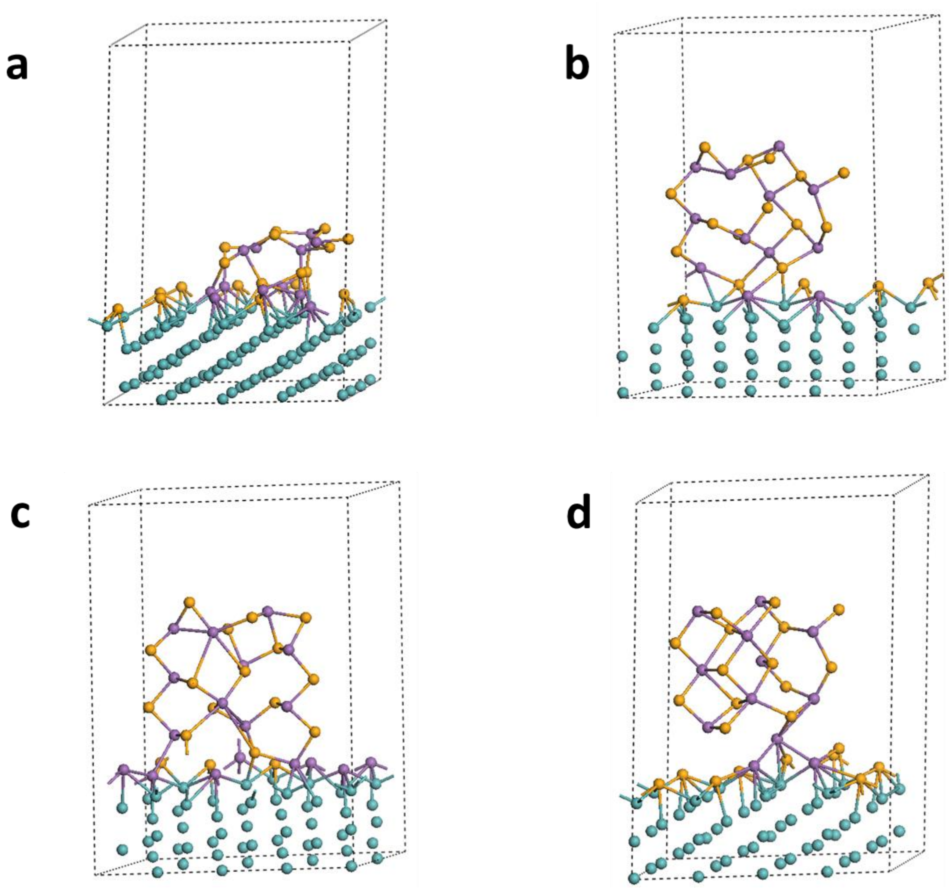

Supplementary Figure 3 Simulated Sb and Se atom distribution for four different interface absorption models. a, (Sb_4Se_6/Mo) Sb_4Se_6 unit parallel to the Mo layer; **b,** (Sb_4Se_6/Mo) Sb_4Se_6 unit perpendicular to the Mo layer; **c,** Sb_4Se_6/Sb/Mo ; **d,** Sb_4Se_6/Se/Mo.

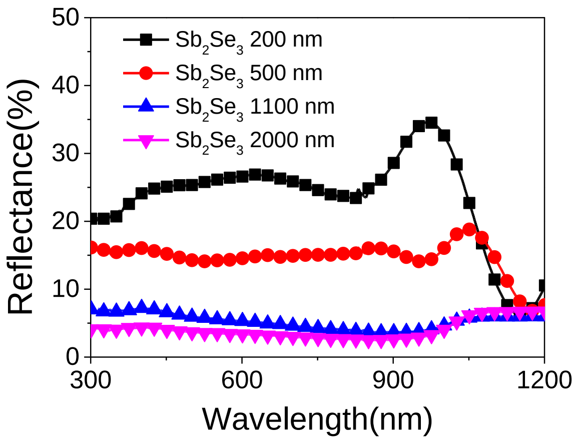

Supplementary Figure 4 Reflectance spectra of Sb_2Se_3 absorber with different growth time from 60s to 180s. The spectral reflectance of the Sb_2Se_3 absorber deposited on Mo substrate with different deposition time 60 s, 120 s, 160 s and 180 s, corresponding to the thickness (length) of 200 nm, 500 nm, 1100 nm and 2000 nm, respectively, as shown in Figure 2. A high reflectance value of around 25 % is observed in the visible wavelength range for the sample grown for 60 s, indicating a smooth surface of the Sb_2Se_3 film with about 200 nm thickness. The reflectance decreases to about 15% for the sample grown for 120 s, which is due to the rough Sb_2Se_3 thin film surface resulting from the nanorod texture. A further increase in growth time led to a more gradual decrease in reflectance. The reflectance approaches 2% for the Sb_2Se_3 nanorod arrays grown for 180 s.

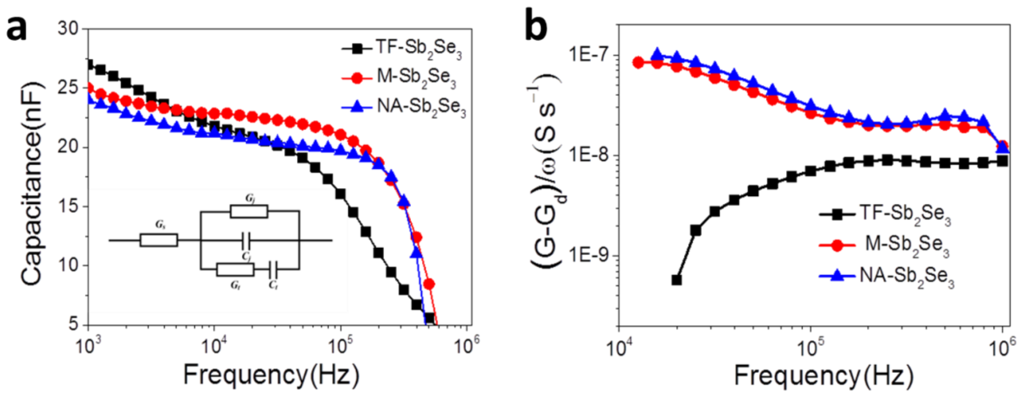

Supplementary Figure 5 a. Frequency dependent capacitance of the solar cells. The inset is AC equivalent circuit model. b. Frequency dependent normalized conductance $(G-G_d)/\omega$.

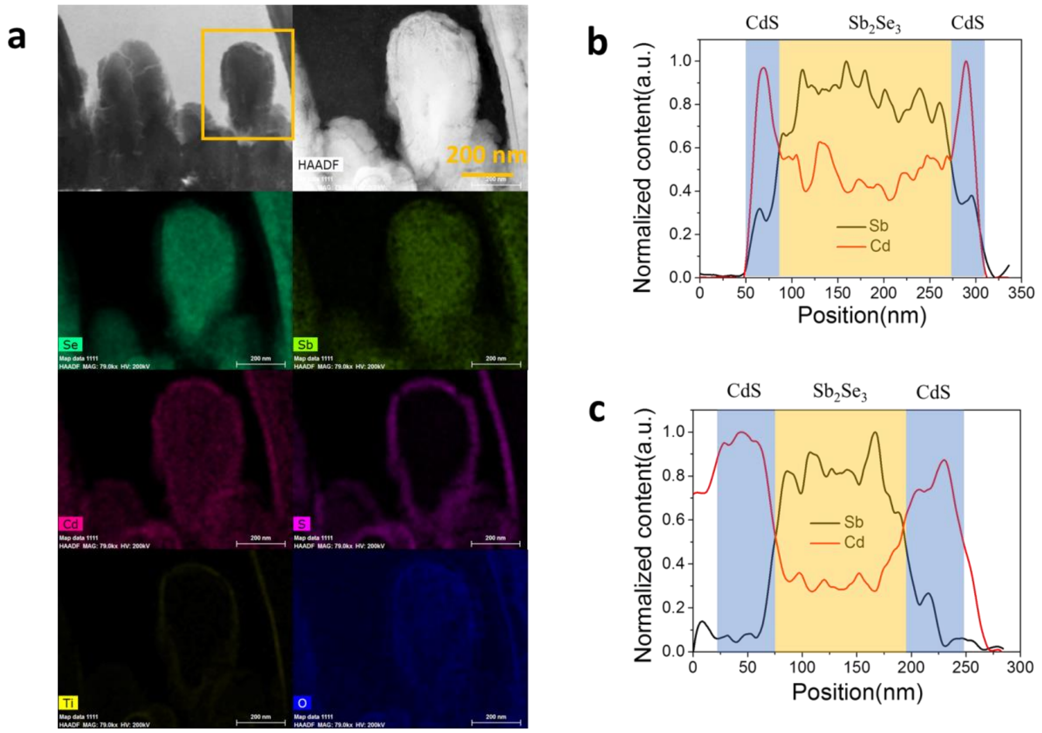

Supplementary Figure 6 HADDF and EDX analysis of the CdS/TiO_2 modified Sb_2Se_3 nanorod. a, HAADF-STEM image and EDX element mapping of the CdS/TiO_2 modified Sb_2Se_3 nanorod.Element detected: Se, Sb, Cd, S, Ti an d O. **b,** EDX line scan of the element Sb and Cd distribution of the CdS/Sb_2Se_3 nanorod. In the CdS/Sb_2Se_3 nanorod, two shoulders in the Sb distribution, located at 30% and 40% relative content, were observed in the CdS shell. **c.** EDX line scan of Element Sb and Cd distribution of the CdS/TiO_2 modified Sb_2Se_3 nanorod. Only one smaller shoulder in Sb distribution, located at 20% relative content, was detected in the CdS shell of the CdS/TiO_2 modified Sb_2Se_3 nanorod.

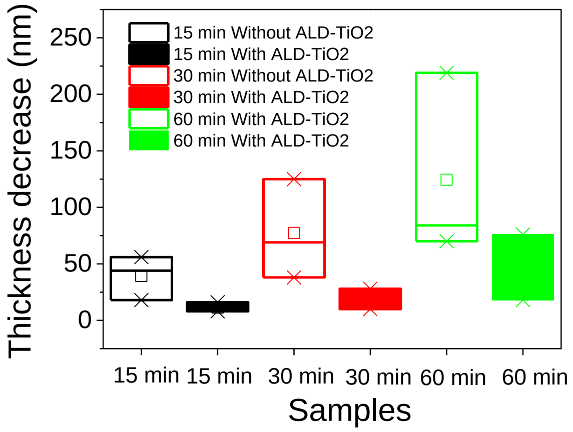

Supplementary Figure 7 The thickness decrease for the samples with and without ALD-TiO_2. It displays the decrease in thickness for the Sb_2Se_3 nanorod array samples with and without ALD-TiO_2 coating. The thickness loss of the ALD-TiO_2 coated samples was obviously smaller than that of the Sb_2Se_3 nanorod array without any coating, suggesting that even a thin ALD-TiO_2 layer, just several nanometer thick, can reduce the dissolution of Sb_2Se_3 nanorod array in the solution of $CdSO_4$ and NH_4OH.

中国计量科学研究院

测试报告

Test Report

证书编号 GXtc2018-1086
Certificate No.

客户名称 Client	河北大学 Hebei University
器具名称 Instrument	硒化锑太阳电池 Sb2Se3 thin film solar cells
型号/规格 Type/Model	Sb2Se3
出厂编号 Serial No.	P2-18061305
生产厂家 Manufacturer	/
客户地址 Address	河北省保定市五四东路 180 号
测试日期 Date of Test	2018-06-15

批准人：
Approved by

中国计量科学研究院 1 测试专用章

地址：中国 北京 北三环东路 18 号
Address：No.18 Bei San Huan Dong Lu,Beijing,P.R.China
邮编：100029
Post Code
电话：+86-10-64525569/74
Tel
传真：+86-10-64271948
Fax
网址：http://www.nim.ac.cn
Website
电子邮箱：kehufuwu@nim.ac.cn
Email

中国计量科学研究院

证书编号 GXtc2018-1086
Certificate No.

中国计量科学研究院是国家最高的计量科学研究中心和国家级法定计量技术机构。1999 年授权签署了国际计量委员会（CIPM）《国家计量基(标)准和国家计量院签发的校准与测量证书互认协议》（CIPM MRA）。The National Institute of Metrology (NIM) is China' s national metrology institute (NMI) and a state-level legal metrology institute. NIM is China' s signatory to the Mutual Recognition of National Measurement Standards and of Calibration and Measurement Certificates Issued by National Metrology Institutes (CIPM MRA) which is arranged by the International Committee of Weights and Measures (CIPM).

中国计量科学研究院的质量管理体系符合 ISO/IEC17025 标准，通过中国合格评定国家认可委员会和亚太计量规划组织（APMP）联合评审的校准和测量能力（CMCs）在国际计量局（BIPM）关键比对数据库中公布。NIM' s quality management system meets requirements of the ISO/IEC 17025. Its Calibration and Measurement Capabilities (CMCs) that are peer reviewed both by China National Accreditation Service for Conformity Assessment (CNAS) and the Asia Pacific Metrology Programme (APMP) are published in the International Bureau of Weights and Measures (BIPM) Key Comparison Database (KCDB).

2011 年，中国计量科学研究院和中国合格评定国家认可委员会就认可领域的技术评价活动签署了谅解备忘录，承认中国计量科学研究院的计量支撑作用和出具的校准/检测结果的溯源效力。NIM and CNAS signed a Memorandum of Understanding (MOU) for Recognition of Technical Assessment in Laboratory Accreditation Field in 2011, in which CNAS recognizing the technical supporting role of NIM in laboratory accreditation and the traceability of NIM's calibration / test results.

测试结果不确定度的评估和表述均符合 JJF1059 系列标准的要求。The evaluation and expression of uncertainty of the test results are in line with the requirements of JJF1059 series standards.

测试所依据的技术文件（代号、名称）Reference documents (Code,Name)
Measurement of photovoltaic current-voltage characteristics（IEC60904-1）
太阳电池校准规范：光电性能（JJF 1622-2017）(Calibration Specification of Solar Cells: Photoelectric Properties)

测试环境条件及地点 Test place and environment
温 度 Temperature：24.8±1 ℃ 地 点 Location： 和平里 5 号楼 401 室
湿 度 Humidity：35±1 %RH 其 它 Others：

测试使用的计量基（标）准装置(含标准物质)/主要仪器
Reference Standards（Including the Reference Material）/ Instruments used

名 称<br>Name	测量范围<br>Measurement Range	不确定度/<br>准确度等级<br>Uncertainty/Accuracy	证书编号<br>Certificate No.	证书有效期至<br>Due Date<br>(YYYY-MM-DD)
太阳电池光电性能校准装置<br>Measurement Standard	I_{sc}:（0.1-10）A<br>V_{oc}: (0.1-200)V<br>P_m:(0.01-500) W	I_{sc}: 1.5%（k=2）<br>V_{oc}: 0.5% (k=2)<br>P_m:1.6% (k=2)	[2015]国量标计证字第 286 号	2019-07-05
标准太阳电池<br>Reference solar cell	I_{sc}:（0-200）mA	1.2% (k=2)	GXtc2018-0439	2019-03-12

第 2 页 共 5 页

第 1 次更改
2018-06-26

中国计量科学研究院

证书编号 GXtc2018-1086
Certificate No.

测试结果
Calibration Results

1. 测试条件 Test Conditions：

标准太阳电池：单晶硅（81#）；

Reference Solar Cell: mono-Si（81#）；

温度传感器/控制系统：无；

Teperature Sensor/Control System: None;

电压设置：0.45V~-0.1V；间隔：-.01 V

Scan Parameter: From 0.45V to -0.1V with -.01 V interval

光阑 Mask（Y/N）: Y

扫描时间： 14 秒　　扫描点数： 56

Scan Time: 14 s　　Scan Point: 56

2. I-V 特性参数 I-V Characteristic parameters:

以上述标准太阳电池标定太阳模拟器辐照度至 1000 W/m^2，校准被测太阳电池的 I-V 特性曲线和参数如下：

By using the above reference solar cell to calibrate the solar simulator's irradiance to 1000 W/m^2, the I-V characterisitc curve and parameters as follows:

扫描方向：正扫

Scan Direction: forward

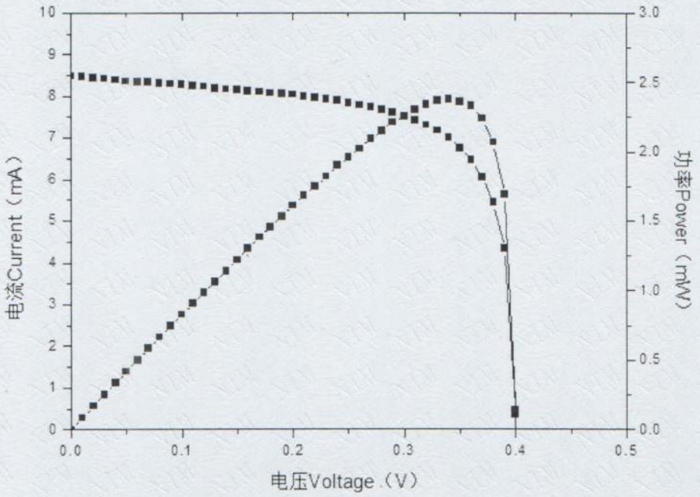

第 3 页　共 5 页

中国计量科学研究院

证书编号 GXtc2018-1086
Certificate No.

测试结果
Calibration Results

有效面积 (mm^2)	短路电流 I_{sc}(mA)	开路电压 V_{oc}(V)	最大功率 P_{max}(mW)
26.023	8.48	0.40	2.38

最大功率电流 I_{max}(mA)	最大功率电压 V_{max}(V)	填充因子 FF (%)	转换效率(PCE) η (%)
7.01	0.34	70.3	9.2

扫描方向：反扫
Scan Direction: reverse

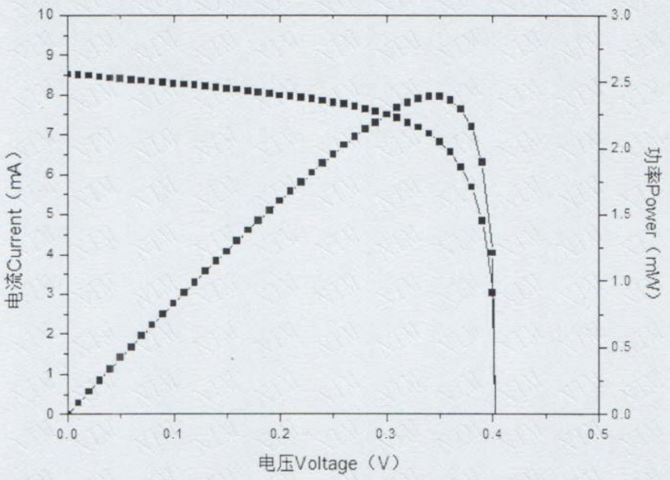

中国计量科学研究院

证书编号 GXtc2018-1086
Certificate No.

测试结果
Calibration Results

有效面积 (mm^2)	短路电流 I_{sc}(mA)	开路电压 V_{oc}(V)	最大功率 P_{max}(mW)
26.023	8.52	0.40	2.39

最大功率电流 I_{max}(mA)	最大功率电压 V_{max}(V)	填充因子 FF (%)	转换效率(PCE) η (%)
7.02	0.34	70.1	9.2

注 Note:

1. 测试所用 mask 的面积为 26.023mm^2（证书编号：CDjc2018-3929）。
The mask area is 26.023mm^2 (Certificate No.: CDjc2018-3929).
2. 此数据仅对被测样品当时状态有效。
The data apply only at the time of the test for the sample.

（以下空白）

声明 Statement:

1.我院仅对加盖“中国计量科学研究院校准专用章”的完整证书负责。
NIM is ONLY responsible for the complete certificate with the calibration stamp of NIM.
2.本证书的测试结果仅对所校准的计量器具有效。
The certificate is ONLY valid for the test ed instrument.
3.本证书用中英文两种语言表达，准确含义以中文为准。
The certificate is reported in both English and Chinese, with the Chinese version as standard.

测 试 员：[illegible]　　核 验 员：[illegible]

第 5 页　共 5 页

Supplementary Figure 8 The certificate of our Sb_2Se_3 solar cell by National institute of Metrology (NIM), China.

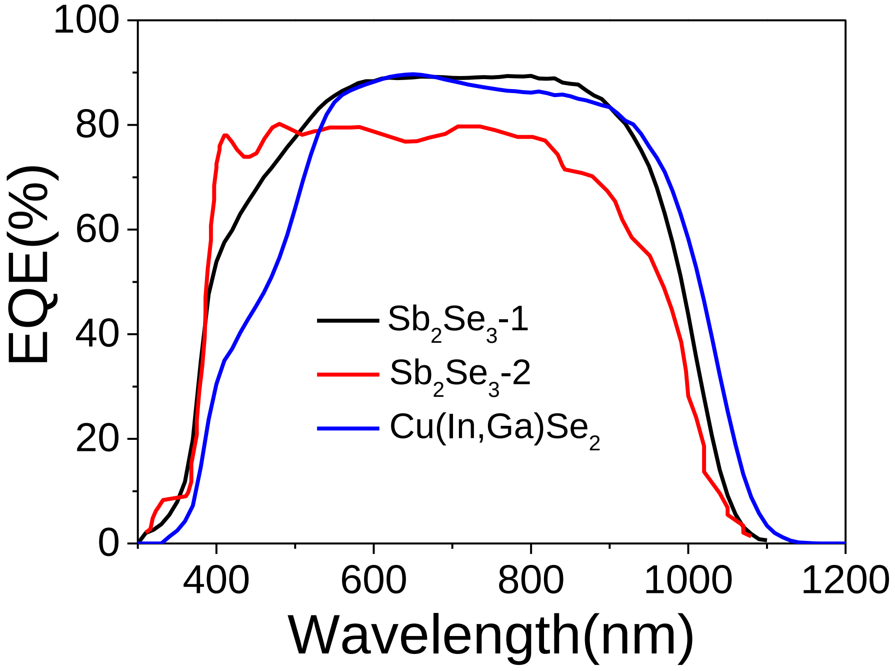

Supplementary Figure 9 Comparison of the external quantum efficiency (EQE) spectra for solar cells with different configurations: Sb_2Se_3-1 is short for AZO/ZnO/CdS/Sb_2Se_3 nanorod arrays/Mo in substrate configuration in this work, Sb_2Se_3-2 is short for FTO/ZnO/Sb_2Se_3 thin films/Au in superstrate configuration [1], and Cu(In,Ga)Se_2 is short for AZO/ZnO/CdS/Cu(In,Ga)Se_2 thin film/Mo in substrate configuration. The integrated current was 31.48, 28.1 and 31.8 mA cm^{-2} for Sb_2Se_3-1, Sb_2Se_3-2 and Cu(In,Ga)Se_2, respectively.

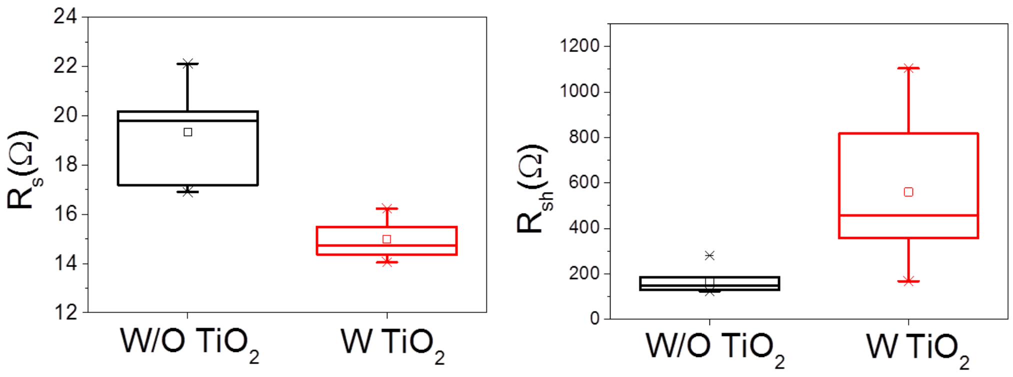

Supplementary Figure 10 The shunt and series resistance for the solar cells with and without TiO_2 modification.

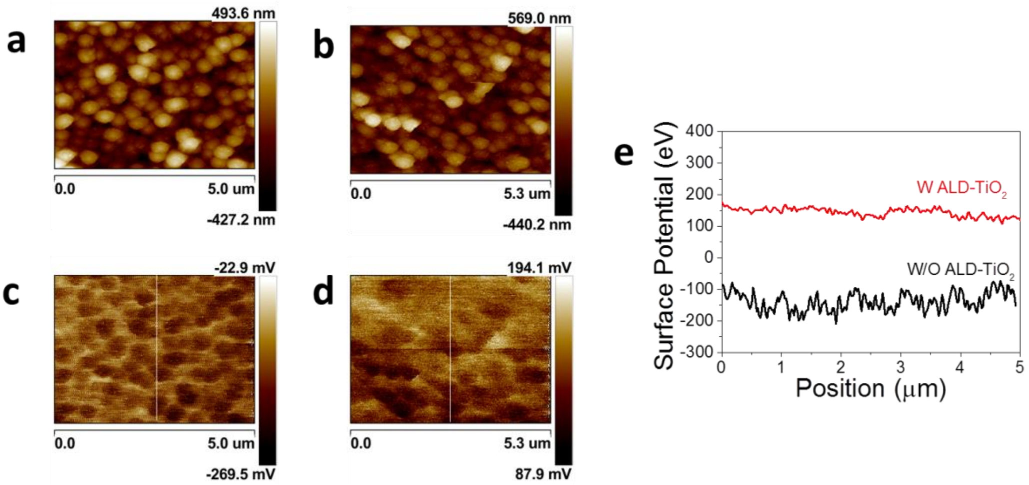

Supplementary Figure 11 a,b Two-dimensional topography of the Sb_2Se_3 nanorod array absorbers without (a) and with (b) ALD-TiO_2. **c,d** Two-dimensional surface potential spatial mappings of the Sb_2Se_3 nanorod array absorbers without and with ALD-TiO_2, respectively. **e,** The profile of the surface potential of the Sb_2Se_3 nanorod array absorbers without and with ALD-TiO_2.

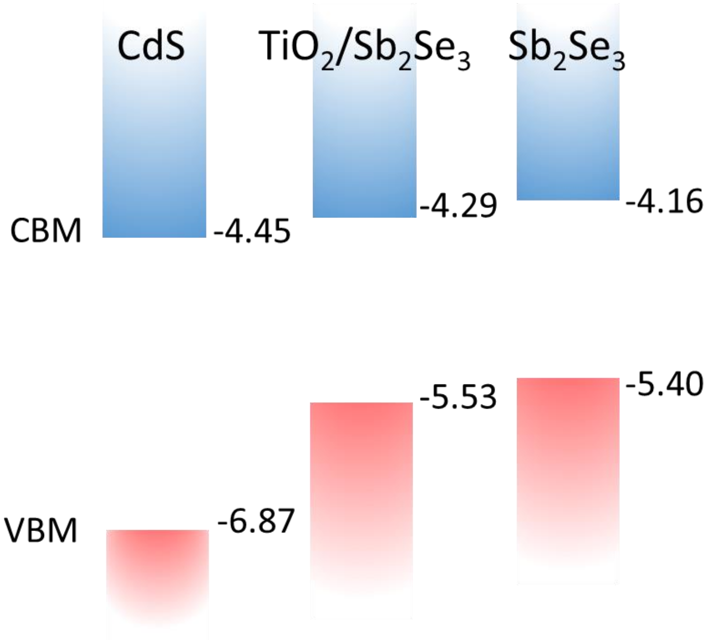

Supplementary Figure 12 Schematic energy band diagram of the CdS, TiO_2-modified Sb_2Se_3 and Sb_2Se_3 layers.

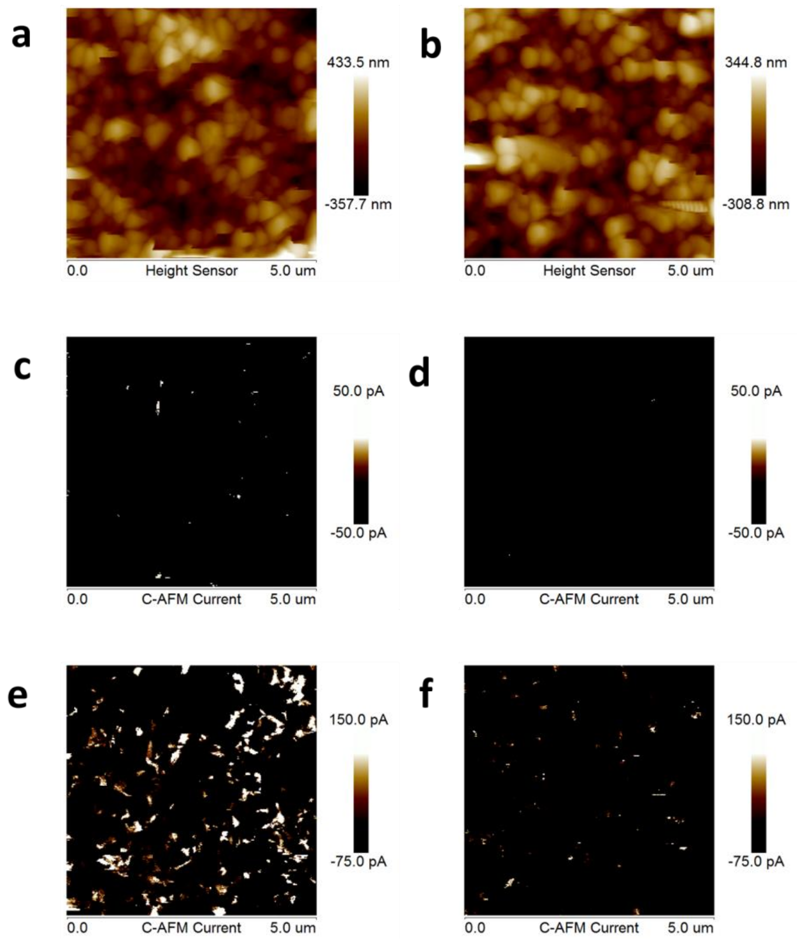

Supplementary Figure 13 Two-dimensional topography and C-AFM images of the CdS/Sb_2Se_3 junctions. a, b Two-dimensional topography of the CdS/Sb_2Se_3 junction without (**a**) and with (**b**) ALD-TiO_2. **c, d** Two-dimensional conductive atomic force microscopy (C-AFM) images under an bias of 1 V for the CdS/Sb_2Se_3 without (c) and with (d) ALD-TiO_2. **e, f** Two-dimensional C-AFM images under an bias of 2 V for the CdS/Sb_2Se_3 without (e) and with (f) ALD-TiO_2.

Supplementary Table 1 Atom displacement for the different absorption interfaces.

Displacement (Å)	Sb4Se6/ Mo(110)	Sb4Se6/Sb /Mo(110)	Sb4Se6/Se /Mo(110)	Sb4Se6/Mo(110) (parallel)
**Se**	0.39519	0.32378	0.45897	1.41368
**Sb**	0.45386	0.32634	0.48822	0.81662
**Sb**	0.49255	0.41527	0.5496	1.03161
**Sb**	0.51545	0.56511	0.68411	1.07744
**Sb**	0.58493	0.57382	0.71153	1.76344
**Sb**	0.68653	0.70991	0.73412	1.0422
**Sb**	0.69112	0.7163	0.80497	1.2638
**Sb**	0.75077	0.72253	0.92786	3.12445
**Sb**	0.75765	0.75741	1.19239	1.16555
**Sb**	0.75865	0.77566	1.23584	0.98155
**Sb**	0.84136	0.80704	1.26142	0.99291
**Sb**	1.01763	0.90899	1.30303	2.92803
**Se**	1.09235	0.94769	1.38322	0.98968
**Se**	1.15297	1.00508	1.3964	3.77627
**Se**	1.15464	1.10477	1.42819	1.83933
**Se**	1.17761	1.12432	1.50495	5.0179
**Se**	1.33397	1.18115	1.52692	1.26073
**Se**	1.42133	1.29899	1.5464	2.98705
**Se**	1.49131	1.56456	1.59128	1.17679
**Se**	1.61025	1.58193	1.63973	1.32712
**Se**	1.62087	1.64567	1.65146	0.65286
**Se**	1.67118	1.66313	1.66	1.56967
**Se**	1.78621	1.70548	1.67086	0.87188
**Se**	1.87969	1.87638	1.67304	0.81646
**Se**	2.02777	1.95501	1.80018	1.43758
**Se**	2.06995	1.96348	1.80039	0.46868
**Se**	2.12838	1.98399	2.06774	0.83524
**Se**	2.14794	2.37872	2.1313	0.82283
**Se**	3.10524	2.43531	2.18788	1.93011
**Se**	3.42198	2.44689	2.58299	3.29179
**Standard deviation**	0.75522095	0.642111	0.534465	1.072486

Supplementary Table 2 The element Sb and Se contents in the solution after 15 min and 2 hours immersion in CBD solution.

Duration time	Element	Content	Unit
2h	Se	0.5463	mg/L
15 min	Se	0.0793	mg/L
2h	Sb	0.7562	mg/L
15 min	Sb	0.0699	mg/L

Supplementary Note 1 Considering the influence of trap levels in the bandgap, the equivalent circuit for the CdS/Sb_2Se_3 hetrojunction solar cells is shown in Supplementary Figure 5a. The measured capacitance includes the contribution of junction and trap levels[2, 3]. Trap levels in the space charge region of a *pn* junction contribute to its admittance as follows[4, 5]: the traps are filled with electrons up to the Fermi level and interact with the nearest band edge by thermal capture and emission of carriers. During the measurement, a small dc voltage is usually applied to the junction to modulate the Fermi level with respect to the band edges and thereby modulate the occupancy of the trap states. Moreover, the filling and emptying of the traps take place at the emission rate, and the amount of trapped charges decreases when the trap occupancy can no longer follow the rapid jitter of the Fermi level; thus the trap capacitance decreases with increasing frequency. To separate the contribution of traps to the conductance G, the dc conductance of traps was subtracted from the measured values.[6] The frequency dependent normalized conductance $(G-G_d)/\omega$ was plot in Supplementary Figure 5b. No obvious peaks could be observed in the medium or high frequency region.

Supplementary Note 2 Two Sb_2Se_3 nanorod array samples were immersed in a pre-heated solution of 1 mM $CdSO_4$ and 0.2 M NH_4OH. The concentration of $CdSO_4$ and NH_4OH was the same as that of the chemical bath deposition (CBD) solution for CdS deposition. The temperature and pH value were 70°C and 10.5, respectively. The samples were immersed for different durations. The content of element Se and Sb in the solution was measured by inductively coupled plasma-atomic emission spectrometry (ICP-AES Agilent ICPOES 730). As shown in Supplementary Table 2, Sb and Se elements are found in the solution just after just 15 minute immersion. The concentrations of the two elements increase by about a factor of ten as the duration was extended to 2h. This indicates that the Sb_2Se_3 nanorod arrays might be dissolved in the standard CBD process.

Supplementary References:

[1] L. Wang, D.-B. Li, K. Li, C. Chen, H.-X. Deng, L. Gao, Y. Zhao, F. Jiang, L. Li, F. Huang, Y. He, H. Song, G. Niu, J. Tang, Stable 6%-efficient Sb_2Se_3 solar cells with a ZnO buffer layer, Nat. Energy, 2 (2017) 17046.

[2] P. Viktorovitch, G. Moddel, Interpretation of the conductance and capacitance frequency dependence of hydrogenated amorphous silicon Schottky barrier diodes, J. Appl. Phys., 51 (1980) 4847-4854.

[3] S. S. Hegedus, W. N. Shafarman, Thin-film solar cells: device measurements and analysis, Prog. Photovolt. Res. Appl., 12 (2004) 155-176.

[4] T. Walter, R. Herberholz, C. Müller, H.W. Schock, Determination of defect distributions from admittance measurements and application to $Cu(In,Ga)Se_2$ based heterojunctions, J. Appl. Phys., 80 (1996) 4411-4420.

[5] M. Luo, M. Leng, X. Liu, J. Chen, C. Chen, S. Qin, J. Tang, Thermal evaporation and characterization of superstrate CdS/Sb_2Se_3 solar cells, Appl. Phys. Lett., 104 (2014) 173904.

[6] J. Kneisel, K. Siemer, I. Luck, D. Braunig, Admittance spectroscopy of efficient CuInS[sub2] thin film solar cells, J. Appl. Phys., 88 (2000) 5474-5481.
