# Supplementary material for: 9.2%-efficient core-shell structured antimony selenide nanorod array solar cells
Source: Nat Commun. 2019 Jan 10;10:125. doi: 10.1038/s41467-018-07903-6 (PMC6328536; doi:10.1038/s41467-018-07903-6)
Supplement: Supplementary file 3 — Reporting Summary [file 41467_2018_7903_MOESM3_ESM.pdf]

nature research

Corresponding author(s): Zhiqiang Li

Solar Cells Reporting Summary

Nature Research wishes to improve the reproducibility of the work that we publish. This form is intended for publication with all accepted papers reporting the characterization of photovoltaic devices and provides structure for consistency and transparency in reporting. Some list items might not apply to an individual manuscript, but all fields must be completed for clarity.

For further information on Nature Research policies, including our data availability policy, see Authors & Referees.

Experimental design

Please check: are the following details reported in the manuscript?

1. Dimensions

Item	Yes	No	Details
Area of the tested solar cells	☒	☐	Figure 6d on Page 9 of the main text
Method used to determine the device area	☒	☐	We used mask to confirm the cell area. Supplementary Figure 8 on page 13 of the Supplementary information

2. Current-voltage characterization

Item	Yes	No	Details
Current density-voltage (J-V) plots in both forward and backward direction	☒	☐	Supplementary Figure 8 on page 11-13 of the Supplementary information
Voltage scan conditions<br>*For instance: scan direction, speed, dwell times*	☒	☐	Supplementary Figure 8 on page 11 of the Supplementary information
Test environment<br>*For instance: characterization temperature, in air or in glove box*	☒	☐	Supplementary Figure 8 on page 10, 11 of the Supplementary information
Protocol for preconditioning of the device before its characterization	☒	☐	Supplementary Figure 8 on page 11 of the Supplementary information
Stability of the J-V characteristic<br>*Verified with time evolution of the maximum power point or with the photocurrent at maximum power point; see ref. 7 for details.*	☒	☐	Figure 7 on page 10 of the main text

3. Hysteresis or any other unusual behaviour

Item	Yes	No	Details
Description of the unusual behaviour observed during the characterization	☐	☒	No hysteresis behaviour was observed
Related experimental data	☐	☒	Supplementary Figure 8 on page 8-13 of the Supplementary information

4. Efficiency

Item	Yes	No	Details
External quantum efficiency (EQE) or incident photons to current efficiency (IPCE)	☒	☐	Figure 4b and 6e
A comparison between the integrated response under the standard reference spectrum and the response measure under the simulator	☒	☐	Page 8-9 of the main text
For tandem solar cells, the bias illumination and bias voltage used for each subcell	☐	☒	N/A

5. Calibration

Item	Yes	No	Details
Light source and reference cell or sensor used for the characterization	☒	☐	Supplementary Figure 8
Confirmation that the reference cell was calibrated and certified	☒	☐	Supplementary Figure 8

Calculation of spectral mismatch between the reference cell and the devices under test

☐ Yes
☒ No

No mismatch calculation was performed

6. Mask/aperture

Size of the mask/aperture used during testing

☒ Yes
☐ No

26.023 mm2, Figure 6d on Page 8-9 of the main text and Supplementary Figure 8

Variation of the measured short-circuit current density with the mask/aperture area

☒ Yes
☐ No

Supplementary Figure 8

7. Performance certification

Identity of the independent certification laboratory that confirmed the photovoltaic performance

☒ Yes
☐ No

Supplementary Figure 8

A copy of any certificate(s)
Provide in Supplementary Information

☒ Yes
☐ No

Supplementary Figure 8

8. Statistics

Number of solar cells tested

☒ Yes
☐ No

Figure 6f on page 8-9

Statistical analysis of the device performance

☒ Yes
☐ No

Figure 6f on page 8-9

9. Long-term stability analysis

Type of analysis, bias conditions and environmental conditions
For instance: illumination type, temperature, atmosphere humidity, encapsulation method, preconditioning temperature

☒ Yes
☐ No

Figure 7 on page 10
